# A case report: Giant cystic parathyroid adenoma presenting with parathyroid crisis after Vitamin D replacement

**DOI:** 10.1186/1472-6823-12-14

**Published:** 2012-07-28

**Authors:** Ali Asghar, Mubasher Ikram, Najmul Islam

**Affiliations:** 1 Section of Endocrinology, Department of Medicine, Aga Khan University Hospital, Stadium Road, P.O. Box 3500, Karachi, 74800, Pakistan; 2 Section of Otorhinolaryngology, Department of Surgery, Aga Khan University Hospital, Stadium Road, P.O. Box 3500, Karachi, 74800, Pakistan

**Keywords:** Parathyroid, Adenoma, Cystic, Hypercalcemia, Hyperparathyroidism, Vitamin D, Crisis, Mediastinum, Hypokalemia, Sestamibi

## Abstract

**Background:**

Parathyroid adenoma with cystic degeneration is a rare cause of primary hyperparathyroidism. The clinical and biochemical presentation may mimic parathyroid carcinoma.

**Case presentation:**

We report the case of a 55 year old lady, who had longstanding history of depression and acid peptic disease. Serum calcium eight months prior to presentation was slightly high, but she was never worked up. She was found to be Vitamin D deficient while being investigated for generalized body aches. A month after she was replaced with Vitamin D, she presented to us with parathyroid crisis. Her corrected serum calcium was 23.0 mg/dL. She had severe gastrointestinal symptoms and acute kidney injury. She had unexplained consistent hypokalemia until surgery. Neck ultrasound and CT scan revealed giant parathyroid cyst extending into the mediastinum. After initial medical management for parathyroid crisis, parathyroid cystic adenoma was surgically excised. Her serum calcium, intact parathyroid hormone, creatinine and potassium levels normalized after surgery.

**Conclusion:**

This case of parathyroid crisis, with very high serum calcium and parathyroid hormone levels, is a rare presentation of parathyroid adenoma with cystic degeneration. This case also highlights that Vitamin D replacement may unmask subclinical hyperparathyroidism. Consistent hypokalemia until surgery merits research into its association with hypercalcemia.

## Background

Primary hyperparathyroidism is an uncommon disease, with a reported incidence of approximately 21 cases per 100,000 persons per year [1]. Solitary parathyroid adenoma accounts for approximately 85% of these cases. Less commonly, it may be caused by multiple adenomas or parathyroid hyperplasia [2]. Rarely, it may be caused by parathyroid carcinoma or parathyroid cyst.

Cystic lesions of the parathyroid gland are very uncommon, accounting for less than 0.01% of all neck masses [3]. Microcyst (<1 cm) is usually found normally in the population and more so in aging parathyroid gland [4]. Macrocyst (≥1 cm) may be of clinical significance and should be evaluated. Most cystic parathyroid adenomas are located in the neck, although around 10% are found in the mediastinum [5]. A parathyroid cyst may arise from vestigial remnants of third or fourth branchial clefts or the Kursteiner canals, or it may be formed by accumulation of fluid in the parathyroid gland (retention cyst) or by degeneration of an adenoma or carcinoma (pseudocyst) [6]. When parathyroid cysts have been identified before biochemical work-up, it has been documented that approximately 10 % are functional [7]; however, when cervical exploration is performed for both hyperparathyroidism or cervical mass, cystic parathyroid lesions are seen in up to 4% of patients [8,9], and functional parathyroid cysts are much more common than non-functional parathyroid cysts [9].

Although majority of patients with cystic parathyroid adenoma present with mild hypercalcemia, some may present with parathyroid crisis [6,10,11]. Parathyroid crisis is a rare life-threatening complication of primary hyperparathyroidism occurring in 1-2% of patients [12]. It is associated with very high serum calcium levels and multi-organ failure. We present the case of a middle aged lady who presented with parathyroid crisis.

## Case presentation

A 55 year old lady had history of depression and acid peptic disease for last eight years. Her serum calcium was 11.4 mg/dL (8.6-10.2) eight months ago, but she was never worked up. She complained of generalized body aches, lethargy and worsening epigastric discomfort for last three months. Her Vitamin D level was found to be <4.0 ng/mL (Vitamin D deficiency: <20). Her general practitioner prescribed her two injections of Vitamin D_3_ 600,000 I.U. IM over a period of two weeks.

Few days after receiving last Vitamin D_3_ injection, she developed increased thirst, increased urinary frequency, reduced appetite, severe nausea, vomiting and constipation. She presented with these complains to our emergency department. On examination, she was awake, alert and oriented, but she was dehydrated. A 3 x 3 cm, firm, non-tender, smooth mass was felt at the lower pole of left lobe of thyroid gland. Her serum calcium was 22.0 mg/dL (8.6-10.2), phosphorus was 2.6 mg/dL (2.5-4.5), albumin was 2.7 g/dL (3.2-5.5), and corrected calcium was 23.0 mg/dL. She had high BUN of 26 mg/dL (6–20), high serum creatinine of 1.4 mg/dL (0.6-1.1), and low serum potassium of 2.9 mmol/L (3.5-5.1). Vitamin D was 119 ng/mL (Vitamin D sufficiency: >30, Vitamin D intoxication >150), Intact PTH (Parathyroid Hormone) level was 1182 pg/mL (16–87) and TSH was 0.88 uIU/mL (0.5-8.9). Serum potassium remained <3.5 mmol/L (3.5-5.1) during hospital stay until surgery was performed, despite being replaced time and again. Her 24-hour urinary calcium was 397 mg (100–300) at corrected serum calcium of 13.94 mg/dL (8.6-10.2); and her 24-hour urinary potassium was 18 mmol (26–123) at serum potassium of 2.6 mmol/L (3.5-5.1). Her symptoms, together with very high serum calcium, high BUN and creatinine, suggested that she was in parathyroid crisis. Her clinical and biochemical profile led to the suspicion of parathyroid carcinoma. She was initially medically managed with intravenous fluids, intravenous Pamidronate and intramuscular Calcitonin.

An ultrasound examination of the neck revealed a large cyst measuring 6.0 x 3.7 cm on the left side appearing to represent the left lobe of thyroid, with thrombosis of left internal jugular vein. A small cyst was also seen at lower pole of right thyroid gland measuring 9 x 7 mm. There were no features to suggest rupture of the cyst.

**Figure 1:**
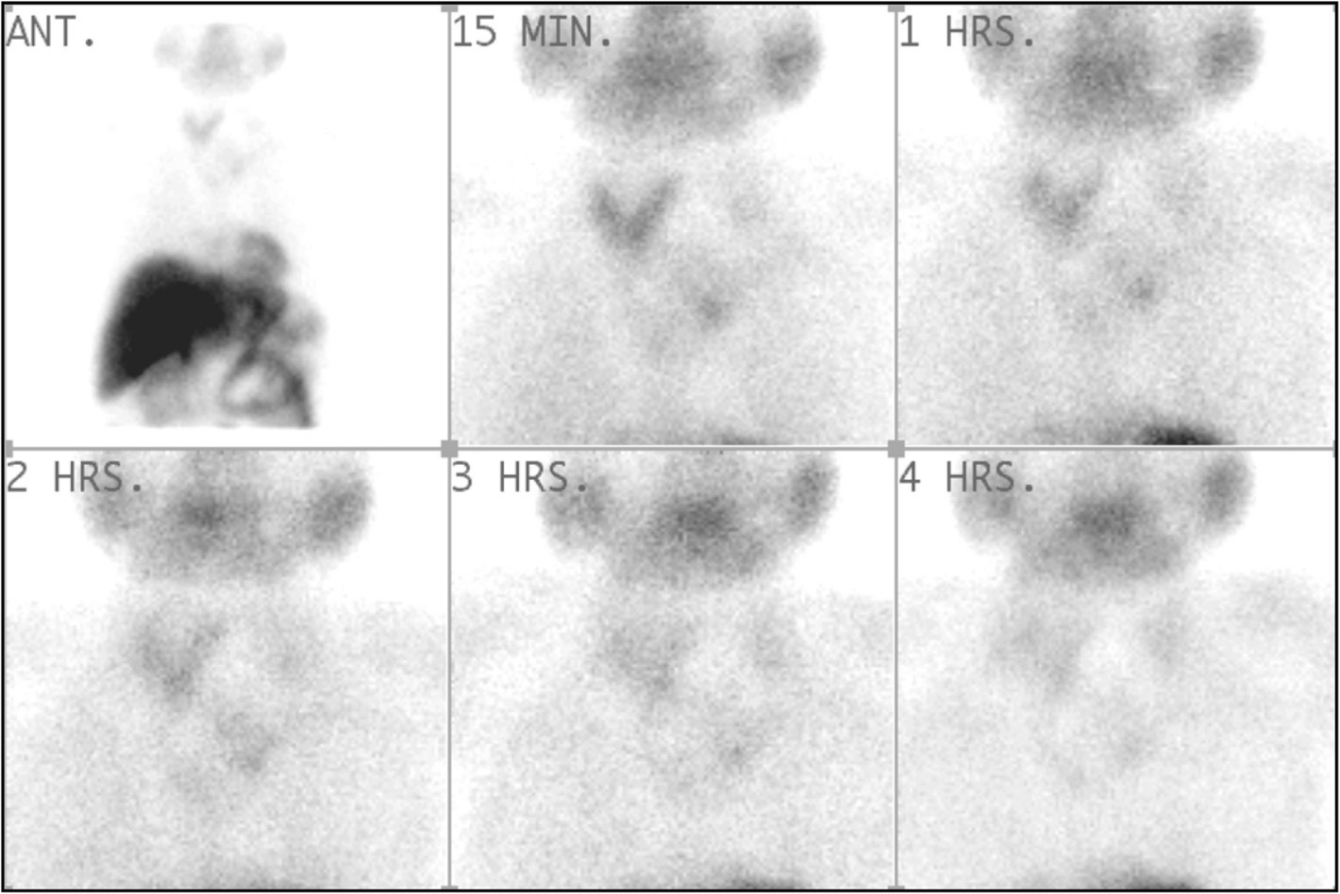
Radionuclide parathyroid scintigraphy showing cystic lesion over left side of neck.

Radionuclide parathyroid scintigraphy, performed with 600 MBq of Tc-99 m sestamibi, revealed a cystic lesion over left side of neck displacing thyroid gland on the right and a superior mediastinal mass with minimal tracer retention (Figure 1).

**Figure 2:**
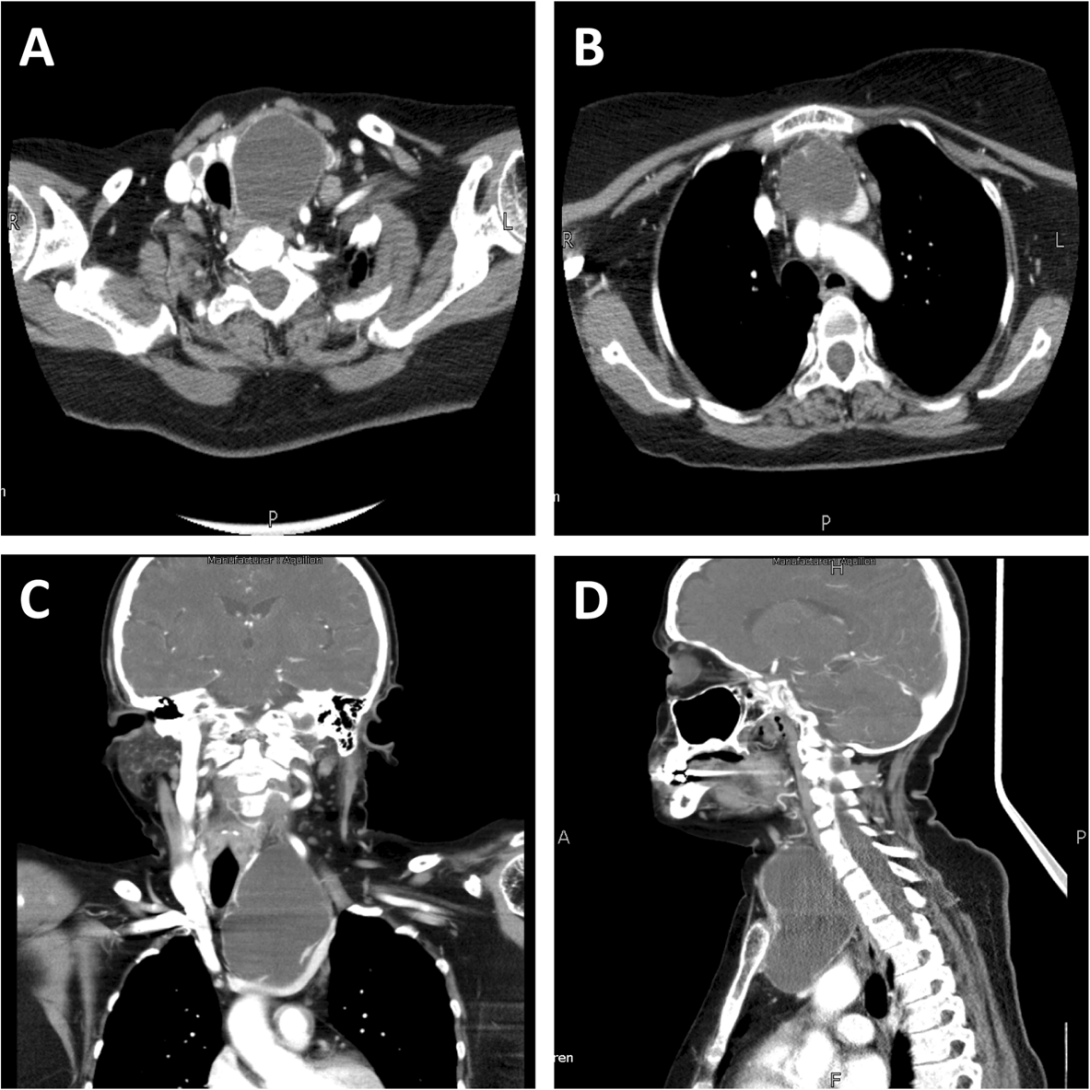
CT scan of head and neck showing parathyroid cystic mass extending into mediastinum.

CT scan of head and neck showed large hypodense lesion in left side of neck with peripherally enhancing walls measuring approximately 5.2 x 4.5 x 8 cm, which appeared to be scalloping the left thyroid lobe and resulting in mass effect with deviation of the trachea to the right. Inferiorly it appeared to be extending into the retrosternal region. Mass effect was also noted on left internal jugular vein with evidence of filling defect representing thrombosis. Small hypodense lesion was also identified in right lobe of thyroid measuring 0.9 x 0.7 cm. Few subcentimeter prevascular lymph nodes were noted. There were no features to suggest rupture of the cystic lesion (Figure 2).

**Figure 3:**
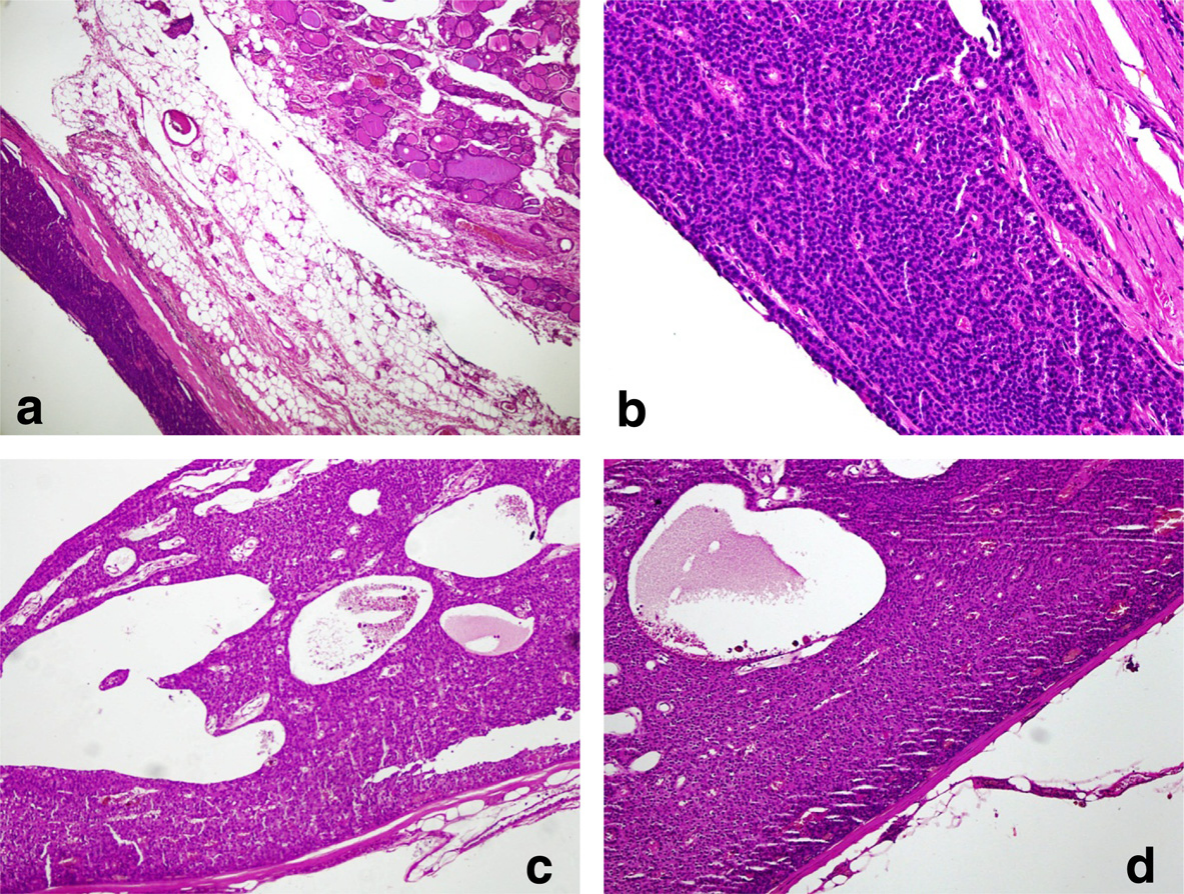
a, b: Parathyroid adenoma lying adjacent to normal thyroid tissue. c, d: Parathyroid adenoma with predominant cystic degeneration.

As the cystic mass was extending into the mediastinum and there was suspicion of parathyroid carcinoma, surgery was jointly done by otorhinolaryngology and thoracic surgery teams in order to attain good surgical clearance. It was a T shaped incision with horizontal part in the neck and vertical part extending onto the chest. A huge cystic mass (11 x 7 x 6 cm), which was adherent to the thyroid gland, was excised along with left lobe of thyroid gland. Ten suspicious looking lymph nodes from levels VI and VII were also removed. Histopathology showed parathyroid adenoma with prominent cystic degeneration with no evidence of metastasis in the lymph nodes (Figure 3).

The clinical and biochemical profile of our patient normalized within a few days after surgery. She was being given 2400 mg elemental calcium supplement daily. One week after surgery, her Intact PTH level was 67.9 pg/mL (16–87), calcium was 7.5 mg/dL (8.6-10.2), albumin was 1.5 g/dL (3.2-5.5), corrected calcium was 9.5 mg/dL, creatinine was 1.2 mg/dL (0.6-1.1), and potassium was 3.5 mmol/L (3.5-5.1). She was continued with daily 2400 mg elemental calcium supplement. A month after surgery, her Intact PTH level was 84.60 pg/mL (16–87), calcium was 7.7 mg/dL (8.6-10.2), albumin was 1.9 g/dL (3.2-5.5), corrected calcium was 9.38 mg/dL, creatinine was 0.9 mg/dL (0.6-1.1), and potassium was 4.2 mmol/L (3.5-5.1). The patient did not have postoperative hypocalcemia, and there were no clinical features to suggest hungry bone syndrome.

## Discussion

This case highlights the rare presentation of parathyroid cystic adenoma extending into the mediastinum presenting with parathyroid crisis and with very high serum calcium and Intact PTH levels. In addition, the size of the cystic adenoma was huge. The clinical and biochemical presentation mimicked that of parathyroid carcinoma. This case also illustrates the unmasking of primary hyperparathyroidism after Vitamin D replacement in Vitamin D deficient patients. The consistent hypokalemia seen in this patient together with hypercalcemia needs further research.

Parathyroid crisis is a life-threatening emergency. It is also known in literature as acute hyperparathyroidism, parathyroid storm, parathyrotoxicosis, acute parathyroid intoxication, parathormone intoxication, hypercalcemic crisis and calcium intoxication. It was first described in 1923 by Dawson and Struthers [13]. It is characterized by severe hypercalcemia (>14 mg/dL or >3.5 mmol/L) associated with signs and symptoms of multi-organ failure [12,14,15]. Although most cases of severe hypercalcemia are seen in malignancy [14], it is important to emphasize that severe hypercalcemia with raised PTH is pathognomonic of primary hyperparathyroidism [16]. Patients with parathyroid crisis demonstrate gastrointestinal and neurological symptoms, renal failure and cardiac rhythm abnormalities. Our patient had very high corrected serum calcium level of 23.0 mg/dL. She exhibited gastrointestinal features like nausea, vomiting and constipation. She was severely dehydrated and had acute kidney injury as confirmed by her laboratory indices. She didn’t have any neurological or cardiac rhythm abnormalities.

The biochemical profile, size of the cystic adenoma, and the fact that it was palpable, all mimicked parathyroid carcinoma. Corrected serum calcium level of 23.0 mg/dL is rarely seen with parathyroid adenoma [17]. Today, primary hyperparathyroidism usually presents with mild hypercalcemia (within 1 mg/dL above the upper limit of normal), is usually asymptomatic, and diagnosed incidentally on routine investigations for minor non-specific complains. Parathyroid carcinoma, on the other hand, presents with severe hypercalcemia (>14 mg/dL) [18]. High serum calcium seen in our patient was associated with a high Intact PTH level (1182 pg/mL). E. Shane suggested that benign disease have PTH level <2 times the upper limit of normal, whereas carcinoma may have PTH level up to 10 times the upper limit of normal [18]. Robert et all suggested that PTH level <4 times the upper limit of normal excludes malignancy [19]. Case series studying solitary parathyroid adenomas with asymptomatic disease derived mean preoperative PTH levels of 186 pg/mL [20] and 165 pg/mL [21], while research studying parathyroid carcinoma yielded higher PTH levels of 714 pg/mL [22] and 1220 pg/mL [23]. The huge size of the cystic adenoma (11 x 7 x 6 cm) in our case is extremely rare [3], though giant parathyroid cysts of up to 15 cm in diameter have also been reported [24]. It may be interesting to note that a palpable neck mass is highly unusual in primary hyperparathyroidism [25]. Hence, parathyroid carcinoma should be borne in mind while dealing with huge, palpable parathyroid masses, with very high serum calcium and PTH levels.

Histopathological report of the excised parathyroid lesion may become extremely important in cases that mimic parathyroid carcinoma. According to Shantz and Castleman, the histological features of parathyroid carcinoma are; 1) uniform sheets of (usually chief) cells arranged in a lobular pattern separated by dense fibrous trabeculae, 2) capsular or vascular invasion, and 3) mitotic figures within tumor parenchymal cells that must be distinguished from endothelial cell mitoses [26]. However, these features have also been reported in parathyroid adenomas [25]. Studies suggest that the overall histological appearance should be taken into consideration, and the presence of more than one in a lesion should raise suspicion of parathyroid carcinoma [27,28]. The histopathology in our case did not show any of these features. Our patient had thrombosed left internal jugular vein, which has also been reported previously with large cystic parathyroid lesions compressing internal jugular vein [3].

Data suggest that most patients presenting with parathyroid crisis have underlying chronic hyperparathyroidism [17]. In our case, the diagnosis of hyperparathyroidism was not made prior to the presentation. But there were clues suggesting chronic hyperparathyroidism in our case. The patient had history of depression and acid peptic disease for last eight years. Both these clinical entities could well have been the manifestations of longstanding hypercalcemia. Moreover, serum calcium checked eight months before presentation was elevated at 11.4 mg/dL (8.6-10.2), but no workup was done at that time. The fact that our patient did not experience any neurological symptoms at a corrected serum calcium level of >20 mg/dL somewhat supports the presence of chronic hyperparathyroidism. However, our patient did not have any features to suggest nephrolithiasis, which could have been present in chronic hyperparathyroidism.

It is important to note that our patient developed parathyroid crisis after receiving vitamin D replacement for documented deficiency (<4.0 ng/mL). Although vitamin D was replaced too quickly in our patient, the repeat vitamin D level (119 ng/mL) was not in toxic range, and as such, the hypercalcemia cannot be solely attributed to vitamin D intoxication. But it shows that vitamin D replacement may unmask subclinical primary hyperparathyroidism. PTH helps in converting vitamin D to its active form (1,25-dihydroxy vitamin D), which in turn increases calcium absorption from the intestines. Hence, primary hyperparathyroidism may have been present in our patient chronically (as we discussed earlier), but vitamin D deficiency did not allow the serum calcium levels to rise very high. As soon as vitamin D was replaced, the patient tipped into parathyroid crisis. But in most cases, Vitamin D replacement does not result in life threatening hypercalcemia.

Patients with parathyroid crisis are initially managed medically with fluid hydration followed by loop diuresis and intravenous bisphosphonates [29]. Medical optimization is followed by early excision [30]. Mortality for patients with parathyroid crisis, who are appropriately managed, is reported to be 2.8% [29]. This is an improvement from 14% mortality reported when emergency surgery used to be done instead of bridging it with medical optimization [30,31]. In our patient, we medically optimized before surgically excising the tumour.

We also observed consistent hypokalemia in our patient together with hypercalcemia, until surgery was performed. Urinary excretion of potassium was also low along with hypokalemia. After surgery, both calcium and potassium levels normalized. In horses, hypercalcemia has been associated with hypokalemia with increased urinary potassium excretion [32]. We could not ascertain the cause of this unexplained hypokalemia in our patient. Further research is warranted to look into possible association of hypercalcemia with hypokalemia.

## Conclusion

We presented a rare case of parathyroid crisis secondary to giant parathyroid cystic adenoma extending into the mediastinum, with very high serum calcium and Intact PTH levels, mimicking a parathyroid carcinoma. Hyperparathyroidism was likely chronic in our patient, but presented in parathyroid crisis after Vitamin D replacement, illustrating the unmasking of subclinical hyperparathyroidism by Vitamin D replacement. Consistent hypokalemia in our patient before surgery warrants further studies to ascertain any possible association with hypercalcemia.

## Consent

Written informed consent was obtained from the patient for publication of this Case report and any accompanying images. A copy of the written consent is available for review by the Series Editor of this journal.

## **Abbreviations**

PTH: Parathyroid hormone.

## Competing interests

The author(s) declare that they have no competing interests.

## Authors’ contributions

AA led the conception and design, acquisition of data, review of literature, and drafted the manuscript. MI reviewed the manuscript. NI gave the concept of research paper, and critically reviewed the manuscript. All authors read and approved the manuscript.

## Authors' information

AA is member of the Royal Colleges of Physicians of the United Kingdom. He is Fellow in Endocrinology, Diabetes & Metabolism, Department of Medicine, Aga Khan University Hospital. He was involved in the medical management of the patient. MI is fellow of the College of Physicians & Surgeons of Pakistan. He is Associate Professor in Otorhinolaryngology, Department of Surgery, Aga Khan University Hospital. He was patient’s primary surgeon. NI is fellow of the Royal College of Physicians of London. He is Professor in Diabetes, Endocrinology & Metabolism, Department of Medicine, Aga Khan University Hospital. He is also the Director, Diabetes, Endocrinology and Metabolism Fellowship Program, Aga Khan University Hospital. He was patient’s primary physician & endocrinologist.

## Pre-publication history

The pre-publication history for this paper can be accessed here:

http://www.biomedcentral.com/1472-6823/12/14/prepub
